# Associations between polygenic loading, psychosis liability, and clozapine use

**DOI:** 10.1192/j.eurpsy.2023.258

**Published:** 2023-07-19

**Authors:** J. Luykx

**Affiliations:** MUMC+, MUMC+, Maastricht

## Abstract

**Introduction:**

Predictors consistently associated with psychosis liability and course of illness in schizophrenia (SCZ) spectrum disorders (SSD), including the need for clozapine treatment, are lacking. Longitudinally ascertained medication use may empower studies examining associations between polygenic risk scores (PRSs) and pharmacotherapy choices.

**Objectives:**

To examine associations between PRS-SCZ loading and groups with different liabilities to SSD: individuals with SSD on clozapine, individuals with SSD on other antipsychotics, their parents and siblings, and unrelated healthy controls; and between PRS-SCZ and the likelihood of receiving a prescription of clozapine relative to other antipsychotics.

**Methods: Design:**

Six-year follow-up and cross-sectional observational cohort study.

**Setting:**

Multi-center.

**Participants:**

Individuals diagnosed with SSD using clozapine or other antipsychotics, their parents and siblings, and unrelated healthy controls.

**Exposure:**

PRS-SCZ.

**Main Outcomes and Measures:**

We used multinomial logistic regression to examine possible differences between groups by computing risk ratios (RRs), i.e., ratios of the probability of pertaining to a particular group divided by the probability of healthy control status. We also computed PRS-informed odd ratios (ORs) for clozapine use relative to other antipsychotics.

**Results:**

PRSs-SCZ were generated for 2344 participants (mean age: 36.95 years; 42.4% female) remaining after quality control (557 individuals with SSD on clozapine, 350 individuals with SSD on other antipsychotics during six-year follow-up, 542 parents and 574 siblings of individuals with SSD, and 321 unrelated healthy controls). All RRs were significantly different from 1; RRs were highest for individuals with SSD on clozapine (RR=3.24 [95%CI 2.76-3.81], p=2.47x10^-46^), followed by individuals with SSD on other antipsychotics (RR=2.30 [95%CI 1.95-2.72], p=3.77x10^-22^), parents (RR=1.44 [95%CI 1.25-1.68], p=1.76x10^-6^), and siblings (RR=1.40 [95%CI 1.21-1.63], p=8.22x10^-6^). PRS-SCZ was positively associated with clozapine versus other antipsychotic use (OR=1.41 [95%CI 1.22-1.63], p=2.98x10^-6^), suggesting a higher likelihood of clozapine prescriptions in individuals with higher PRS-SCZ.

**Conclusions:**

PRS-SCZ loading differs between groups of individuals with SSD, their relatives, and unrelated healthy controls, with clozapine users being at the far end of PRS-SCZ loading. Additionally, PRS-SCZ is associated with a higher likelihood of clozapine prescribing. Our findings may inform early intervention and prognostic studies into the value of PRS-SCZ for personalized antipsychotic treatment.

**Disclosure of Interest:**

None Declared

